# The regulation of cGAS-STING signaling by RNA virus-derived components

**DOI:** 10.1186/s12985-024-02359-1

**Published:** 2024-05-01

**Authors:** Feiting Xie, Qiugang Zhu

**Affiliations:** 1 Zhejiang Key Laboratory of Precision Diagnosis and Therapy for Major Gynecological Diseases, Women’s Hospital, Zhejiang University School of Medicine, 310006 Hangzhou, China; 2 Department of Laboratory Medicine, Shangyu People’s Hospital of Shaoxing, Shaoxing, China

**Keywords:** cGAS, STING, Signaling pathway, RNA virus-derived components

## Abstract

The Cyclic GMP-AMP synthase-stimulator of interferon genes (cGAS-STING) serves as a key innate immune signaling axis involved in the regulation of various human diseases. It has been found that cGAS-STING pathway can recognize a variety of cytosolic double-stranded DNA (dsDNA), contributing to cause a robust type I interferon response thereby affecting the occurrence and progression of viral infection. Accumulating evidence indicates RNA virus-derived components play an important role in regulating cGAS-STING signaling, either as protective or pathogenic factors in the pathogenesis of diseases. Thus, a comprehensive understanding of the function of RNA virus-derived components in regulating cGAS-STING signaling will provide insights into developing novel therapies. Here, we review the existing literature on cGAS-STING pathway regulated by RNA virus-derived components to propose insights into pharmacologic strategies targeting the cGAS-STING pathway.

## Introduction

RNA viruses are responsible for many infectious diseases, including influenza virus, hepatitis C virus, polio, and severe acute respiratory syndrome coronavirus 2 (SARS-CoV-2) [1]. The virus genome continues to mutate, which results in changes in viral infectivity and pathogenicity, creating an escape from antibody as well as vaccine protection thereby posing a greater risk [2]. The main symptoms of virus infection are characterized by fever, cough, fatigue and so on. In severe cases, acute respiratory distress, shock and multi-organ failure may even occur [3, 4]. The innate immune system, as the body’s first line of immune defense, can non-specifically recognize viral pathogen-associated molecular patterns (PAMPs) and launch a signaling cascade that produce proinflammatory cytokines and chemokines [5]. These inflammatory factors could further trigger the cytokine storm, posing a threat to the life of patients. 

The Cyclic GMP-AMP synthase-stimulator of interferon genes (cGAS-STING) pathway serves as a critical mechanism for detecting both endogenous or exogenous DNA, thereby eliciting innate immune responses [6]. cGAS is capable of identifying cytoplasmic DNA anomalies and catalyzing the synthesis of cyclic GMP-AMP (cGAMP) from GTP and ATP. Subsequently, cGAMP binds to and activates the endoplasmic reticulum protein STING, then STING transduces signals to the nucleus through downstream molecules, ultimately leading to the transcription of type I interferons (IFN-I) and interferon-stimulator genes (ISGs), which play an important role in antiviral response [6, 7]. Previous studies have proposed the involvement of the cGAS-STING signaling pathway in RNA virus infections, thereby influencing disease progression [8–10]. In this paper, we primarily delve into the RNA virus-derived components regulate the cGAS-STING signaling pathway and explore the therapeutic potential of targeting this pathway in virus infections.

## Overview of cGAS-STING signaling

cGAS, functioning as an innate immune sensor, possesses the capability to recognize a diverse array of cytosolic dsDNA, originating from viruses, bacteria, mitochondria, and micronuclei, which can be primarily categorized into pathogen-derived DNA and self-DNA [11, 12]. In addition to intracellular dsDNA and dsRNA, cytoplasmic RNA: DNA hybrids can directly activate cGAS, eliciting an effective antiviral immune response [13]. In instances such as simian virus 40 infection, slight DNA damage leakage fosters GAS recruitment and activation [14]. cGAS can also be involved in the detection of tissue damage/formation of DNA traps. After phagocytosis of neutrophil extracellular traps (NETs) by peripheral blood mononuclear cells (PBMCs), their DNA is transferred to the cytoplasm and cGAS is activated in the cytoplasm. Evidence of NETs activation of cGAS in vivo was also obtained in a model of autoimmune hepatitis induced by injection of the lectin concanavalin A [15, 16].

**Fig. 1:**
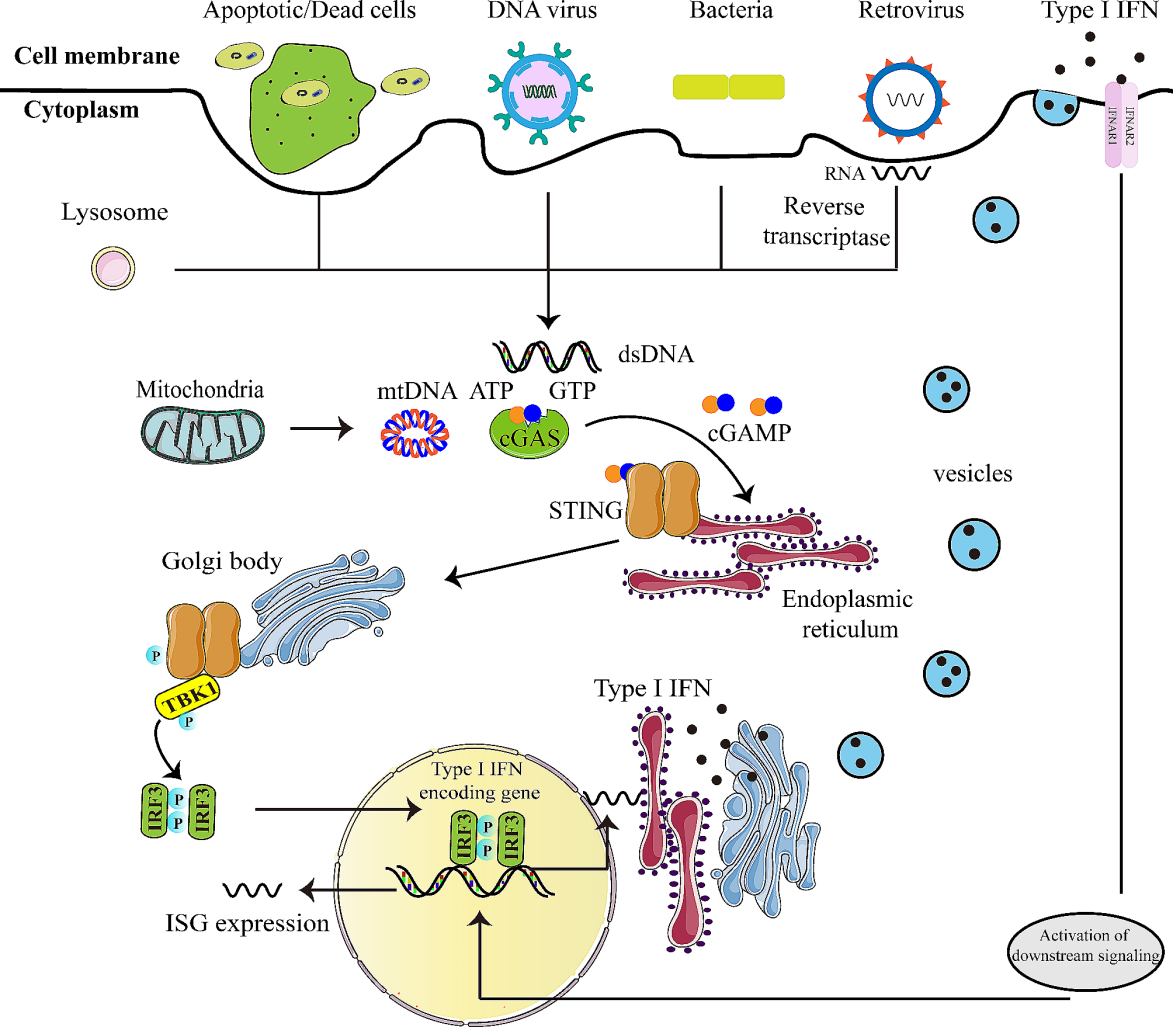
Schematic diagram of cGAS-STING signaling pathway. (cGAS, as an innate immune sensor, is able to recognize various cytoplasmic dsDNA from pathogens, apoptotic/dead cells, mitochondria, and others. The interaction between cGAS and dsDNA leads to enzymatic activation of cGAS and catalyzing the formation of 2’,3’-cyclic GMP-AMP (cGAMP) from ATP and GTP. cGAMP binds to the dimer of interferon gene stimulatory factor (STING) located on the endoplasmic reticulum (ER) membrane. Then, STING moves from the ER to Golgi body via the ER–Golgi intermediate compartment, and then serves as a signaling platform for TBK1 phosphorylation. TBK1 phosphorylates the C-terminal domains of STING, and then IRF3 is recruited and phosphorylated. Finally, dimerized IRF3 can act as a transcription factor to initiate the transcription of type-I IFN and subsequent induction of ISG expression, eliciting antiviral defense)

STING, discovered before cGAS, plays a critical role in DNA recognition and TLR9-independent IFN production [17–19]. In the cytoplasm, cGAS undergoes activation through interaction with dsDNA, a process independent of DNA sequence but reliant on DNA length [20, 21]. Structural analyses have unveiled a distinctive zinc thumb in cGAS responsible for recognizing B-form dsDNA [22]. Activated cGAS catalyzed the ATP and GTP into 2′3′-cyclic GMP-AMP (2′3′-cGAMP) [23]. Then cGAMP bound to and activated STING in the endoplasmic reticulum, promoting tetramer formation of STING through the oligomerization and translocated to the ER-Golgi intermediate compartments [24, 25]. Afterwards, TANK-binding kinase 1 (TBK1) and interferon regulatory factor 3 (IRF3) are recruited by STING, followed by TBK1 autophosphorylation along with phosphorylation of STING and IRF3. The phosphorylated IRF3 dimerizes and localized to the nucleus to initiate IFN-I expression and subsequent induction of ISG expression, thereby instigating antiviral defense (Fig. 1) [6, 26]. Simultaneously, IRF7 and nuclear factor (NF-κB) are activated by TBK1, leading to the expression of other inflammatory cytokines [27, 28]. Notably, STING is also capable of directly detecting viral particles independent of cGAS [29].

## Regulation of cGAS-STING signaling by virus-derived components

Though most studies focused on its role in DNA virus infection, increasing evidence has confirmed that cGAS-STING signaling also participated in RNA virus infection [30, 31]. As a self-protective mechanism, multiple RNA virus-derived components have been reported to be involved in the regulation of cGAS-STING signaling, such as SARS-CoV-2-derived open reading frames, and non-structural proteins, HIV-1-derived Vif, and HCoV-NL63-derived Papin-like protease (PLP) [32–35]. Thus, we will discuss the role of viral components in regulating cGAS-STING signaling in the following sections (Table 1).

**Table 1:** Roles of RNA virus-derived components in cGAS-STING signaling

Components	Source of components	Roles in cGAS-STING signaling	Mechanisms	References	
Spike	SARS-CoV-2	Activating	Inducing cell fusion and damaging nuclei	[8, 46]	
			Inducing cGAS-STING-mediated NF-κB activation	[91]	
ORF3a	SARS-CoV-2	Inhibiting	Binding STING and blocking the nuclear accumulation of p65 to inhibit NF-κB signaling	[48]	
			Disrupting STING-LC3 interaction	[34]	
			Inhibiting cGAS-STING-mediated autophagy	[92]	
ORF9b	SARS-CoV-2	Inhibiting	Interacting with STING and impeding IRF3 phosphorylation and nuclear translocation	[47]	
ORF10	SARS-CoV-2	Inhibiting	Interacting with STING, inhibiting STING-TBK1 interaction and STING ER-Golgi translocation	[9]	
			Impairing STING-mediated autophagy	[9]	
3CL	SARS-CoV-2	Inhibiting	Inhibiting K63-ubiquitin modification of STING (required for NF-κB activation)	[48, 93]	
Vif	HIV	Inhibiting	Inhibiting K63-linked ubiquitination of STING and reduced the production of IFN-I	[32]	
Vpx	HIV	Inhibiting	Suppressing cGAS-STING-mediated NF-κB signaling	[94]	
Vpr	HIV	Inhibiting	Interfering with the degradation of IκBα to suppress cGAS-STING mediated NF-κB signaling	[57]	
Vpu	HIV	Inhibiting	Disrupting the recruitment of IκBβ to hinder the the nuclear translocation of p65	[58]	
NS4B	HCV	Inhibiting	Impairing STING-TBK1 interaction to silence the interferon signaling	[60]	
			Targeting STING and abrogating RIG-I-mediated IFN-I response	[61]	
NS2B3	ZIKV	Inhibiting	In a protease cleavage-dependent manner	[64]	
	DENV		Cleaving cGAS in the N-terminal region	[95]	
NS2B	DENV	Inhibiting	Targeting the cGAS for lysosomal degradation	[75]	
PLP	SARS-CoV-2	Inhibiting	Binding STING and inhibited its ubiquitination	[50]	
	Coronavirus		In an autophagy-dependent manner	[81]	

### cGAS-STING signaling in SARS-CoV-2 infection

SARS-CoV-2 inhibits the expression of IFN-I at the early stage of infection to counteract the IFN-I-mediated innate immune responses through a variety of mechanisms [36–38]. In the early stages of infection, patients with severe COVID-19 exhibited a weak IFN-I response but with a hyper-inflammatory profile, revealing a distinctive and inappropriate inflammatory response [39–41]. However, large but delayed IFN-I responses have also been reported in COVID-19 patients and animal models [42–44]. cGAS-STING signaling, regulated by different components of SARS-CoV-2, is a pivotal pathway involved in the IFN-I production.

#### SARS-CoV-2 activates cGAS-STING signaling

Upon SARS-CoV-2 infection, the production of 2′3′-cGAMP and phosphorylation of STING at Ser366 were hallmarks of cGAS-STING signaling pathway activation [31]. Consistent with the above, SARS-CoV-2 spike protein has been found to induce the expression of IFN-I and ISGs with the cooperation of host proteases [8, 45]. During viral infection-induced syncytium formation in angiotensin-converting enzyme 2-expressing cells, spike proteins damage the nucleus through DNA damage responses, contributing to the formation of micronuclei. Then the micronuclei were sensed by cGAS which localized to the fused cells, subsequently leading to the activation of STING-IRF3 signaling and production of IFN-I [8, 31, 46].

#### SARS-CoV-2 suppresses cGAS-STING signaling

In addition to spike protein, other components of SARS-CoV-2 have been found to inhibit the activation of cGAS-STING signaling [9, 34, 47, 48]. Open reading frame 10 (ORF10), an accessory protein of SARS-CoV-2, has been reported to interfere with STING-TBK1 interaction and inhibit STING ER-Golgi translocation to suppress IFN activation [9]. In addition, ORF10 antagonized the anti-viral response in a STING-mediated autophagy way. Further exploration also found that ORF9b co-localized and associated with STING and TBK-1, reducing the phosphorylation of TBK-1 and IRF3 as well as the nuclear translocation of IRF3, thereby antagonizing IFN-I production [47]. ORF3a, another accessory protein, has been revealed to block cGAS-STING-mediated IFN-β promoter activity in a NF-κB-dependent way [34, 48]. Post-translational modifications play a key role in regulating cGAS-STING signaling [49]. As a protease of SARS-CoV-2, 3CL could inhibit cGAS-STING-mediated NF-κB signaling via suppressing K63-linked ubiquitination of STING [48]. Another component of SARS-CoV-2, PLP, removed K63-linked polyubiquitin chains of STING, thereby disrupting the STING-IKKε-IRF3 complex for the production of IFN-β and ISGs, consequently inhibiting the IFN-I-mediated anti-viral responses [50]. The balance of dual roles in both promoting and suppressing cGAS-STING signaling in SARS-CoV-2 infection still remains to be investigated.

### cGAS-STING signaling in HIV infection

HIV infection leads to progressive CD4^+^ T-cell loss and immune dysfunction, resulting in an increased risk of infections and tumor [30, 51]. Type I interferons are well-characterized innate antiviral proteins that contribute to resistance to HIV-1 infection [52]. Studies have shown that HIV-1 infection induces the cGAS-STING-TBK1-IRF3 signaling pathway which activates innate immunity to produce IFN-I [30, 53]. In recent years, several lines of evidence revealed that cGAS-STING signaling could be regulated by the components of HIV [32, 54]. Src homology 2 (SH2) domain-containing protein tyrosine phosphatase 1 (SHP-1), a protein tyrosine phosphatase, is comprised of two SH2 domains (N-SH2 and C-SH2) and a catalytic domain [55]. A recent study revealed that SHP-1 (residues 243–595) bound to STING (residues 1–137) and thereby inhibited the K63-linked ubiquitination of STING at Lys337 by dephosphorylating STING at Tyr162, thereby reducing the production of IFN-I [32]. This study also pointed out that HIV infection enhanced the inhibitory effect of SHP-1 on STING activation. Mechanistically, HIV-1-derived viral infectivity factor (Vif) promoted the recruitment of SHP-1 to STING and enhanced their interaction, which enhanced SHP-1-mediated inhibition of STING phosphorylation and K63-linked ubiquitination, accompanied by the inhibition of STING oligomerization and the interaction between STING and TBK1, consequently downregulating the IFN-I expression [32]. Another study reported that HIV-2 Vpx (a naturally immunogenic virion-associated protein) suppressed cGAS-STING-mediated NF-κB signaling to promote viral infection [54]. At the same time, Vpx markedly inhibited cGAS-STING-triggered DC maturation, which may have contributed to immune silencing [56]. Vpr and Vpu interact with STING to selectively inhibit NF-ĸB signaling by interfering with the degradation of IκBα and the recruitment of IκBβ [57, 58]. As HIV is cunning enough to evade cGAS-STING-mediated antiviral immune responses in disguise, limiting the inhibitory ingredient of the virus to ‘defenders’ has become a promising therapeutic strategy [32, 54].

### cGAS-STING signaling in HCV infection

Hepatitis C virus (HCV) is a significant pathogen that causes chronic hepatitis liver cirrhosis, and hepatocellular carcinoma worldwide [59]. Ding Q et al. found that activation of the STING-mediated innate immune response to produce IFNs and cytokines inhibited replication of the HCV genotype 1b/Con1 replicon in Huh7.5 cells. STING was crucial for HCV PAMP-induced interferon activation [60]. However, HCV-derived NS4B was found to resist cGAMP stimulation and inhibit STING accumulation, thereby blocking the production of IFN-I and pro-inflammatory cytokines [60, 61]. NS4B silences interferon signaling by disrupting the collaboration between STING and TBK1, and NS4B and NS3/4A may synergistically inhibit different steps of IFN signaling during HCV infection [60].Another study found that NS4B targeted STING and abrogated RIG-I-mediated IFN-I response [62]. To sum up, disruption of these interactions mentioned above may restore IFN-I-mediated antiviral responses and may shed some light on the emergence of novel therapeutic strategies for HCV infection.

### cGAS-STING signaling in ZIKV infection

Zika virus (ZIKV) is a flavivirus transmitted by mosquitoes that can cause significant neurological diseases [63]. According to published data, ZIKV has host tropism and can impair agonist-induced cGAS-STING signaling activation after infecting human cells but not in rodents [64–67]. Specifically, ZIKV blocks the anti-viral function of human STING (hSTING) not only through protease-dependent non-structural protein 2B3 (NS2B3) cleavage, but also potentially through NS2B3 protease cleavage-dependent mechanisms (increased permissiveness) [64]. Recent studies in *Drosophila* have revealed that insect STING homologues exert anti-viral activity against ZIKV infection by inducing autophagy in the brain [65, 67]. Liu et al. demonstrated that ZIKV infection led to the activation of NF-κB signaling, which in turn induces the expression of Drosophila STING (dSTING) in the Drosophila brain [67]. Mechanistically, they claimed that NF-κB-dependent dSTING-dependent autophagy controls ZIKV infection [67]. Overall, the cGAS-STING signaling pathway in restricted ZIKV infection has been well summarized in several papers [67–69].

### cGAS-STING signaling in DENV infection

Dengue fever is a vector-borne viral disease caused by dengue virus (DENV), which evades host “pursuit” by expressing proteins that antagonize cellular innate immunity [70, 71]. DENV has been shown to manipulate cGAS-STING-mediated innate immunity through protein hydrolysis of STING and activation or degradation of cGAS [72–75]. Aguirre et al. demonstrated, for the first time, a clear mechanism of cGAS-STING activation in RNA virus infection [75]. Their study found that the DENV NS2B3 protease complex targeted cGAS for lysosomal degradation to avoid mtDNA sensing, which inhibited IFN-I expression and weakened the antiviral response [75]. A recent study showed that the DENV protease NS2B3 cleaves cGAS in the N-terminal region without destroying the C-terminal catalytic center, resulting in an N-terminal cleavage product (CP-N) and a C-terminal cleavage product (CP-C, including the catalytic center) [72]. Interestingly, the authors found that the DNA-binding affinity of CP-C was lower than that of cGAS, which was also associated with reduced CP-C enzyme activity. In contrast, the DNA binding affinity of CP-N was comparable to that of cGAS. Thus, CP-N competitively inhibited cGAMP production by both cGAS and CP-C [72]. Besides, this study revealed the physical interaction of NS2B3 with cGAS and CP-C, setting the stage for their degradation [72]. Another study revealed the dual role of STING in response to DENV infection [76]. On the one hand, replication of DENV2, a DENV mutant, destroys host DNA, which induces cGAS-STING signaling and the IFN-I response, inhibiting the spread of infection [76]. On the other hand, STING activation also supports DENV2 replication in infected cells through STING-induced autophagy [76].

### Regulation of cGAS-STING signaling by other RNA virus components

It is worth noting that the cGAS-STING signaling can also be regulated by other RNA virus components, such as influenza virus (IAV), encephalomyocarditis virus (EMCV), and lymphocytic choriomeningitis virus (LCMV) [73, 77–79]. In IAV infection, researchers have demonstrated that STING-dependent IFN-β gene expression was indispensable for limiting viral replication. The influenza virus M2 or EMCV 2B protein triggered mtDNA release to initiate cGAS-STING-dependent anti-viral signaling to restrict disease, whereas the influenza virus NS1 binds to mtDNA to attenuate innate immunity [80]. Papain-like proteases (PLP), an important component of human coronavirus (HCoV) NL63 and SARS-CoV, have been reported to antagonize the STING signaling [33, 81]. PLP inhibited STING-mediated IRF-3 nuclear translocation and induction of IRF-3 dependent promoters [33]. Another study found that PLP could regulate STING-mediated innate immune response in an autophagy-dependent manner [81]. PLP2-TM interacted with the key autophagy regulators, LC3 and Beclin1, and promoted Beclin1 interaction with STING. Additionally, knockdown of Beclin1 partially reversed the inhibitory effect of PLP2-TM on innate immune responses [81]. This process was dependent on the interaction between PLP and STING, which blocked dimerization of STING and inhibited the assembly of STING-MAVS-TBK1/IKKe complexes required for activation of IRF-3 [33]. With the deepening of research, cGAS-STING signaling pathway has become a non-negligible presence in the treatment of RNA virus infections.

## Potential therapy based on cGAS-STING signaling

Ongoing clinical trials of cGAS-STING signaling pathway primarily focused on antitumor immunity, whereas, due to growing evidence tapping into the potential of anti-viral immunity, making it a new strategy for the treatment of infections. Considering the pivotal roles of STING in viral infection, several studies have explored the function of STING agonists in therapy [31, 82–84]. Diaminobenzimidazole (diABZI), a small-molecule STING agonist that induces rapid short-term activation of STING, has been reported to inhibit viral replication in infected cells (∼ 1000-fold inhibition) in an IFN-dependent manner [82]. Similar phenomena have been revealed in studies of other STING agonists. Given the inhibitory effect of SARS-CoV-2-dereived 3CL on STING, 3CL inhibitors (such as flavonoids and PF-00835231) have been widely used in the treatment of COVID-19 [85, 86]. Also, it might be promising in the treatment of other 3CL-containing virus-induced infection [87]. However, not all cases of cGAS-STING activation lead to ameliorating the symptoms. Researchers have found that excessive activation of cGAS-STING can exacerbate the virus-induced inflammatory factor storm, which may correlate with the poor prognosis of patients [88]. It has been shown that in different RNA virus-infected cells, immunological differences between viruses can result in different regulatory mechanisms of cGAS-STING. In a study by Christopher J Neufeldt et al., it was found that SARS-CoV-2 infection leads to a storm of inflammatory factors through selective activation of the cGAS-STING signaling axis and thus NF-κB. Inflammatory gene activation was reduced by 60–75% with STING inhibitors [88]. Similar phenomena have been observed in other plus-stranded RNA viruses such as flaviviruses, SARS-CoV and NL63 coronavirus [89]. Application of H-151, a STING inhibitor, attenuates SARS-CoV-2-induced severe lung inflammation and improves disease prognosis [10]. An alternative inhibitor of TBK1/IKKε signaling that disrupts TBK1/IKKε signaling and prevents phosphorylation of S172, thereby blocking IRF3 and STING-mediated NF-κB-mediated transcriptional programs, shows strength in limiting excessive inflammation in SARS-CoV-2 [90]. Thus, the exploration and improvement of STING agonists and protease inhibitors and how to avoid pathological activation of STING during treatment may be the future orientation of treatment. In addition, studies have shown that the cGAS-STING signaling pathway is closely related to a variety of diseases such as tumors, autoimmune diseases, cardiovascular diseases, metabolic diseases, and neurodegenerative diseases, and has great potential to enhance tumor immunity and improve diseases. Therefore, guiding the development of novel targeted drugs for cGAS-STING signaling pathway and taking it into account the safety and efficacy of diseases are also imminent.

## Conclusion

Extensive researches have shed light on the scope and significance of cGAS-STING in antiviral immunity. With its dual role in inhibiting infection dissemination induced by the IFN-I response and promoting viral replication via STING-induced autophagy, STING plays a dual role in response to virus infection. In addition to stimulating IRF3-IFN-I signaling, STING strengthened the transcriptional activity of NF-κB to coordinate innate and adaptive immunity. This review aims to outline the role of components from different RNA viruses in the cGAS-STING signaling pathway, aiming to provide a sound theoretical basis for further studies on viral camouflage to evade cGAS-STING-mediated antiviral immune responses. Studies revealed that activation of STING signaling during RNA virus infection is regulated by a variety of components, such as spike-induced activation and ORF10, ORF3a, ORF9b, 3CL, Vif, and PLP, among others. These components affected cGAS-STING signaling pathway-mediated antiviral responses in IRF-3-dependent, NF-κB-dependent, and autophagy-dependent manner. With the emergence of STING antagonists, therapeutic means to block viral immune evasion have become possible. Furthermore, the interference of accessoryprotein and non-structural protein (NSP, NSP13/14/15) with the production of IFN-I presents new therapeutic avenues for infection. As an important target in antiviral immunity and tumor immunotherapy, it is also imperative to guide the development of novel targeted drugs against the cGAS-STING signaling pathway.

## Data Availability

No datasets were generated or analysed during the current study.

## Abbreviations

cGAS: Cyclic GMP-AMP synthase
STING: Stimulator of interferon genes
dsDNA: Double-stranded DNA
SARS-CoV-2: Severe acute respiratory syndrome coronavirus 2
PAMPs: Pathogen-associated molecular patterns
cGAMP: Cyclic GMP-AMP
ISGs: Interferon-stimulator genes
TLR9: Toll-like receptor 9
TBK1: TANK-binding kinase 1
IRF3: Interferon regulatory factor 3
PLP: Papin-like protease
ORF: Open reading frame
Vif: Viral infectivity factor
SHP-1: Src homology 2 domain-containing protein tyrosine phosphatase 1
HCV: Hepatitis C virus
ZIKV: Zika virus
DENV: Dengue virus
IAV: Influenza virus
EMCV: Encephalomyocarditis virus
LCMV: Lymphocytic choriomeningitis virus
HCoV: Human coronavirus
diABZI: Diaminobenzimidazole
NSP: Non-structural protein
